# Transforming growth factor beta 3 involved in the pathogenesis of synovial chondromatosis of temporomandibular joint

**DOI:** 10.1038/srep08843

**Published:** 2015-03-06

**Authors:** Yingjie Li, Loaye Abdelaziz El.Mozen, Hengxing Cai, Wei Fang, Qinggong Meng, Jian Li, Mohong Deng, Xing Long

**Affiliations:** 1 Department of Oral and Maxillofacial Surgery, The State Key Laboratory Breeding Base of Basic Science of Stomatology & Key Laboratory of Oral Biomedicine Ministry of Education, School & Hospital of Stomatology, Wuhan University, Wuhan, Hubei, China; 2 Department of Orthodontics, School & Hospital of Stomatology, Wuhan University, Wuhan, Hubei, China

## Abstract

Synovial chondromatosis (SC) of temporomandibular joint is rare proliferative disorder featured by the formation of cartilaginous nodules in synovium and joint space. Transforming growth factor beta 3 (TGF-β3) is closely related to chondrogenic differentiation, and might participate in pathogenesis of SC. We discovered that increased quantity of synoviocytes and blood vessels were observed in SC synovium. The vessel wall and sublining fibroblasts were stained positively by the antibodies against TGF-β3, fibroblast growth factor 2 (FGF-2), and CD34. In loose bodies (LBs), TGF-β3 was mainly expressed in chondrocytes and FGF-2 was expressed in chondrocytes, fibroblasts, and vessel walls. Expressions of *TGF-β1*, *TGF-β3*, *FGF-2*, *Sox9*, *Wnt-4*, *Foxc2*, and *VEGF-A* mRNA were significantly higher in SC synovium. Stimulation of TGF-β3 on synoviocytes increased alkaline phosphatase (ALP) activity and expressions of chondrogenic genes (*Sox9*, *Col2α1*, *Aggrecan*, *Wnt-4*, and *Wnt-11*), osteogenic genes (*Runx2*, *Foxc2*, *osteocalcin*, and *Col1α1*), and *VEGF-A*, but failed to influence *FGF-2* expression. However, the addition of FGF-2 increased *TGF-β3* expression. In conclusion, TGF-β3 existed in synovium and LBs of SC, and was responsible for the pathogenesis of SC.

Synovial chondromatosis (SC) of temporomandibular joint (TMJ) is a rare proliferative disorder of synovium accompanied by formation of cartilaginous nodules in synovium and joint space^1^,^2^,^3^, as well as secondary calcification and ossification^4^. The clinical manifestations include unilateral pain, swelling, clicking, occlusal changes, crepitation, and limited mandibular function^5^,^6^.

Transforming growth factor β3 (TGF-β3) was reported to be a potent mediator for inducing chondrogenesis of mesenchymal stem cells (MSCs)^7^,^8^,^9^, and to increase the production of cartilaginous extracellular matrix (ECM)^7^,^10^. The synoviocyte in synovium of TMJ is recognized as MSC because of its potentials to differentiate into adipocyte, chondrocyte, and osteocyte lineages^11^,^12^. Therefore, we hypothesized that TGF-β3 might be responsible for chondrogenic differentiation of TMJ synoviocytes and the pathogenesis of SC.

Besides, our group previously discovered that fibroblast growth factor 2 (FGF-2) was responsible for the formation of loose bodies (LBs) and increased blood vessels of synovium. The relation between TGF-β3 and FGF-2 was therefore investigated in this research.

The main purpose of this study was to investigate the roles of TGF-β3 in the formation of LBs and its relation with FGF-2.

## Results

### Histological and immunohistochemistry (IHC) observations

**Figure 1:**
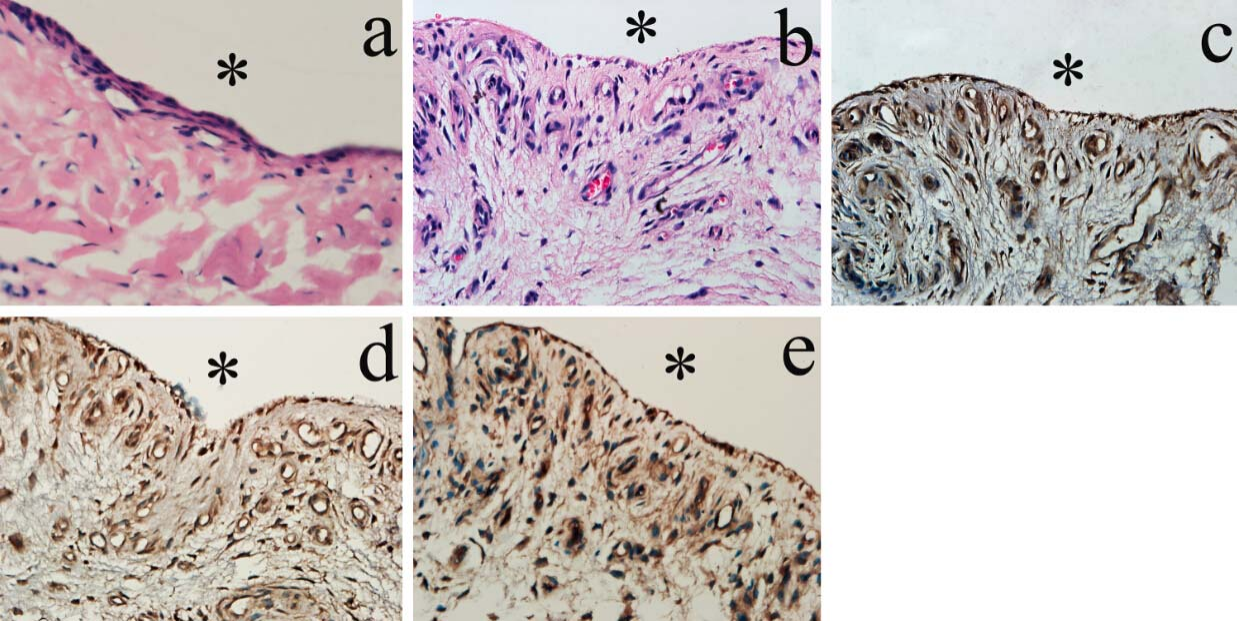
Histological and IHC observations for TMJ synovium facing the articular cavity. HE staining for control synovium (a) and SC synovium (b). IHC observation of TGF-β3 (c), FGF-2 (d), and CD34 (e) in SC synovium. Control synovium was featured by layers of fibroblast-like synoviocytes located in the lining layer and a few fibroblasts and blood vessels in the sublining layer. The SC synovium was characterized by increased amount of fibroblasts and blood vessels in the sublining layer. The vessels wall and sublining fibroblasts were stained positively by the antibodies against TGF-β3, FGF-2, and CD34. * represents for articular cavity. Scale bars: (a~e): 50 μm.

In the normal synovium facing the TMJ articular cavity, fibroblast-like synoviocytes were distributed in form of 3~4 layers in the lining layer. In the sublining layer, a few fibroblasts and blood vessels were observed (Fig. 1a). However, the SC synovium was characterized by increased quantity of fibroblasts and blood vessels in the sublining layer (Fig. 1b). The lining layer of the SC synovium was featured by single and discontinued layer of synoviocytes (Fig. 1b). By IHC examination, the vessels wall and sublining fibroblasts were stained positively by antibodies against TGF-β3 (Fig. 1c), FGF-2 (Fig. 1d), and CD34 (Fig. 1e).

**Figure 2:**
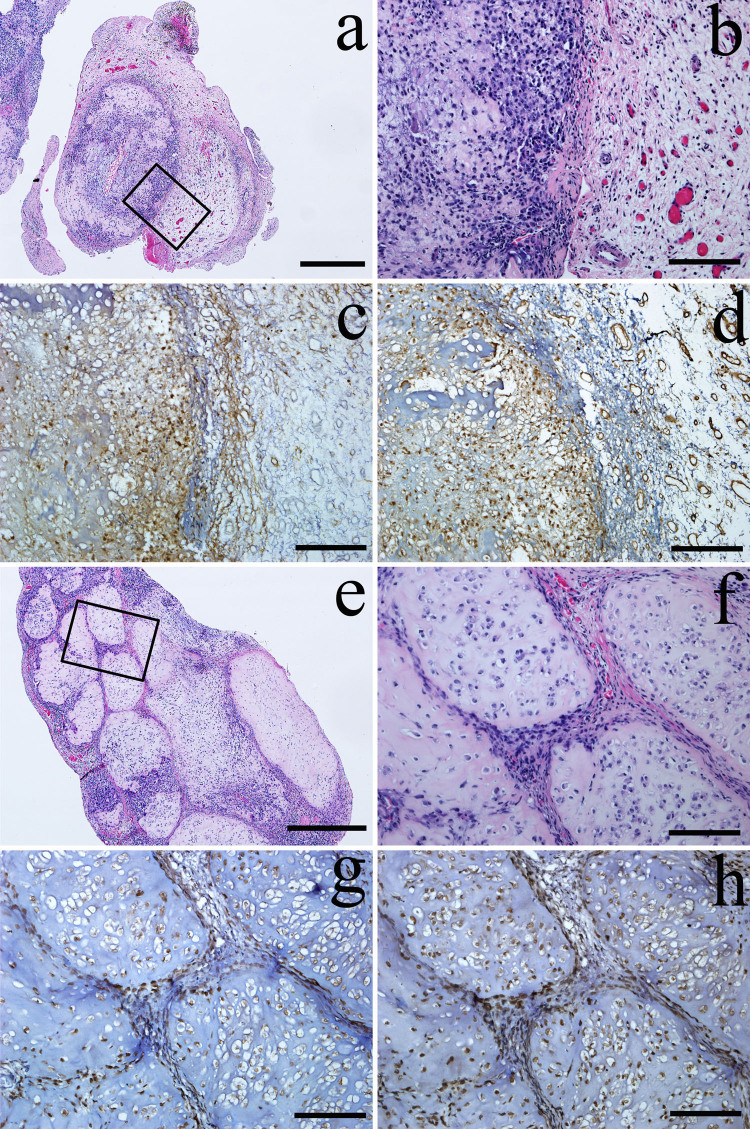
Histological and IHC observations for two types of LBs. One type was the single cartilaginous nodule (a–d), and the other type was numerous nodules composing a large nodule (e–h). In the first type, a thick synovium containing increased quantity of synoviocytes and bloods vessels covered the LB (a and b). In the second type, a connective tissue containing blood vessels and fibroblasts separated these small nodules (e and f). TGF-β3 was expressed mainly in chondrocytes of LBs (c and g) while the FGF-2 was expressed in both chondrocytes and the wall of blood vessels (d and h). b and f are the amplification of the rectangle in a and e, respectively. Scale bars: (a and e): 400 μm; (b~d) and (f~h): 100 μm.

During the surgery, cartilaginous nodules which are also called LBs were observed in the synovium and articular cavity of TMJ. Most of the LBs were too stiff to be chopped by scissors. Two types of LBs were found based upon histological features. LB of the first type was composed of single cartilaginous nodule (Fig. 2a–d). The second type LB was formed by a number of small cartilaginous nodules (Fig. 2e–h). In the first type, a thick synovium which contained increased amount of synoviocytes and blood vessels was found covering the LB (Fig. 2a and b). In the second type, a connective tissue containing small blood vessels and fibroblasts was found to separate the numerous cartilaginous nodules (Fig. 2e and f). TGF-β3 was expressed mainly in the chondrocytes (Fig. 2c and g). FGF-2 was found in chondrocytes of LB (Fig. 2d and h), vessel wall of the synovium (Fig. 2d) and fibroblasts of the connective tissue (Fig. 2h).

### Reverse transcription PCR (RT-PCR) for analyzing SC and normal synovium

**Figure 3:**
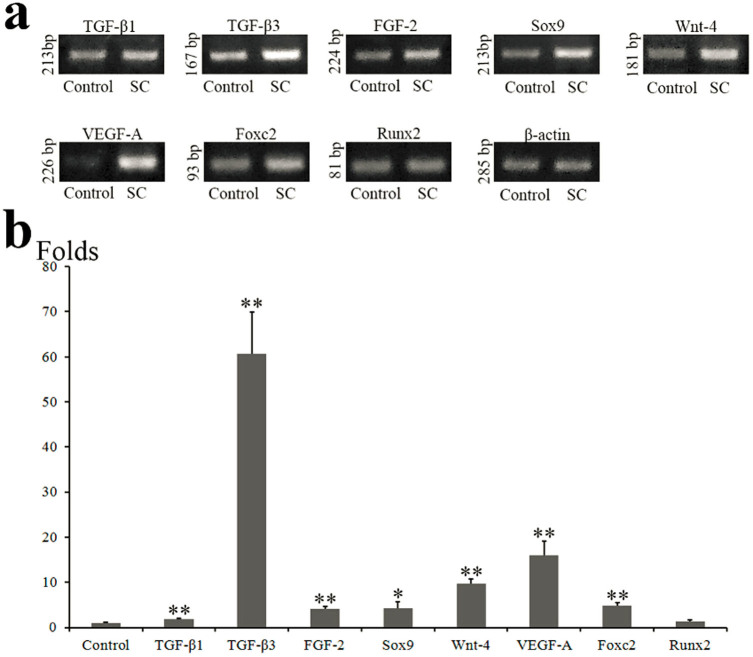
RT-PCR (a) and semi-quantitative evaluation (b) for analyzing the genes expressions in control and SC synovium. *P < 0.05, **P < 0.01 with respect to the control. Semi-quantitative values are presented as mean ± standard deviation of target gene/β-actin. The assay was performed in triplicate under the same experiment conditions. Full-length gels are presented in Supplementary Figure 1.

The expressions of *TGF-β1* and *TGF-β3* in SC synovium were 1.8 and 60.7 times that of the normal synovium (Fig. 3), respectively. In regard to chondrogenic genes, the mRNA expressions of *Sox9* and *Wnt-4* in SC synovium were 4.3 and 9.7 times that of the control (Fig. 3), respectively. In regard to osteogenic genes, *Foxc2* expression in SC synovium was 4.7 times that of the control (Fig. 3). However, the *Runx2* expressions between SC and control synovium had no difference significantly (Fig. 3). The expressions of *FGF-2* and *vascular endothelial growth factor A* (*VEGF-A*) in the SC synovium were 4.1 and 15.9 times that of the control (Fig. 3), respectively.

### The effects of TGF-β3 stimulation on SC synoviocytes

**Figure 4:**
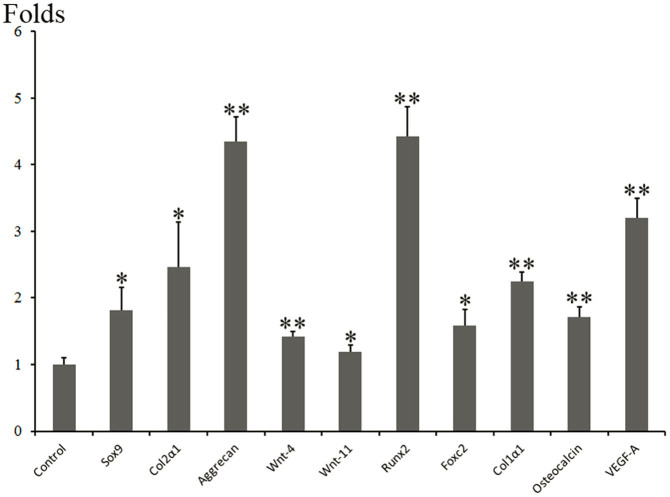
Realtime PCR for analyzing the genes expressions of SC synoviocytes cultured with or without TGF-β3 for 6 days. *P < 0.05, **P < 0.01 with respect to the control. The assay was performed in triplicate under the same experiment conditions.

Compared with the control group, the supplement of TGF-β3 to SC synoviocytes increased the chondrogenic gene expressions of *Sox9*, *Col2α1*, *Aggrecan*, *Wnt-4*, and *Wnt-11* mRNA over 1.8, 2.5, 4.3, 1.4 and 1.29-fold (Fig. 4), respectively. Besides, the osteogenic gene expressions of *Runx2*, *Foxc2*, *Col1α1*, and *osteocalcin* were increased over 4.4, 1.6, 2.3, 1.7-fold (Fig. 4), respectively. *VEGF-A* expression was also increased by 3.2-fold.

**Figure 5:**
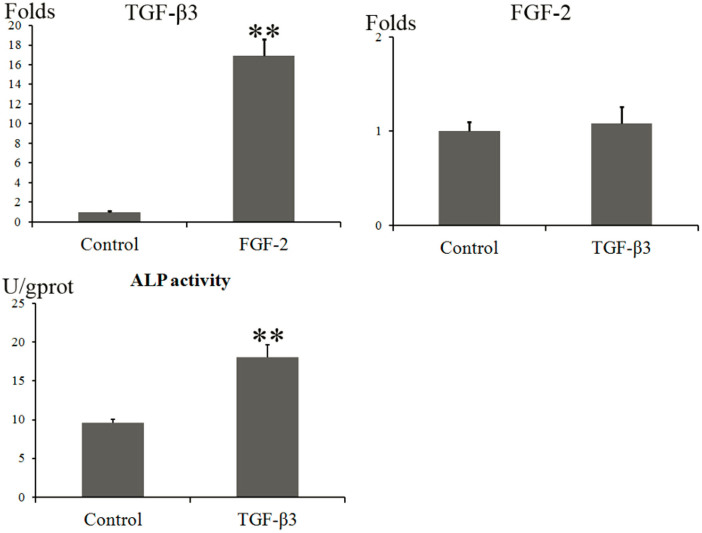
Realtime PCR for determining the interaction between TGF-β3 and FGF-2, and the ALP activity assay. The addition of FGF-2 in the culture medium for SC synoviocytes increased the *TGF-β3* gene expression by 16.9-fold. However, the addition of TGF-β3 did not influence the *FGF-2* expression significantly. The ALP activities in the two groups with and without the addition of TGF-β3 were 18.1 and 9.6 U/gprot, respectively. The assay was performed in triplicate under the same experiment conditions.

The addition of FGF-2 in the culture medium for SC synoviocytes increased the *TGF-β3* gene expression by 16.9-fold (Fig. 5). However, the addition of TGF-β3 did not influence the *FGF-2* expression significantly (Fig. 5). The alkaline phosphatase (ALP) activities in the two groups with and without the addition of TGF-β3 were 18.1 and 9.6 U/gprot (Fig. 5), respectively.

## Discussion

As the TMJ SC is a rare disease, most studies in literature are case report. Basic investigations of SC focus mainly on certain cytokines or proteins in synovium and LBs^1^,^6^,^13^,^14^,^15^ through analysis of IHC staining methods. Comprehensive studies on this disease are hard to find.

This study focused on the role of TGF-β3 in the pathogenesis of SC, because TGF-β3 was potent mediator for inducing chondrogenesis of MSCs^7^,^8^,^16^ and had a higher chondrogenic potential of a more rapid differentiation than TGF-β1^17^. Besides, the cartilaginous nodules were observed in both synovium and joint space^4^,^5^,^18^, and made of cartilaginous ECM and chondrocytes which are absent in normal synovium^19^,^20^. Therefore, we speculated that TGF-β3 might exist in the synovium and LBs. In this study, the existence of TGF-β3 was confirmed by the IHC assay (Fig. 1c, Fig. 2c and g) and the RT-PCR for synovium of SC (Fig. 3).

Then this study examined the effects that TGF-β3 exerted on SC synoviocytes. First, TGF-β3 induced chondrogenic differentiation of SC synoviocytes, supported by the increased levels of mRNA expressions of *Sox9*, *Col2α1*, *aggrecan*, *Wnt-4* and *Wnt-11* in this study (Fig. 4). *Sox9* is a potent mediator of chondrocyte phenotype, and regulates the expressions of key chondrogenic genes including *Col2α1*, *Col11α2*, *Col9α1*, and *aggrecan*^21^,^22^.

Besides, TGF-β3 elevated the expressions of osteogenic genes, such as *Runx2*, *Foxc2*, *Col1α1* and *osteocalcin* in this study (Fig. 4). Foxc2 is involved in regulating both osteogenesis and angiogenesis of MSCs^23^. The angiogenic effect of Foxc2 might explain partly for the phenomenon of increased angiogenesis in synovium of SC (Fig. 1b, Fig. 2b). Runx2 is a common target of bone morphogenetic protein-2 (BMP-2) and TGF-β1, and plays an essential role in osteogenic differentiation^24^. BMP-2 was reported to participate in the pathogenesis of cartilaginous and osteogenic metaplasia in SC^25^. However, the *Runx2* expression had no statistical difference between SC and control synovium (Fig. 3), a phenomenon which could be explained by our group's previous finding^26^ that the stimulation of FGF-2 on SC synoviocytes down-regulated the *Runx2* expression. Therefore, the integrated effect of FGF-2 and TGF-β3 caused no statistical difference in *Runx2* expression between SC and control synovium.

Moreover, TGF-β3 upregulated the expression of angiogenic gene *VEGF-A*. Previously, we found that the addition of FGF-2 to the medium for SC synoviocytes also upregulated the expression of *VEGF-A*^26^. Therefore, both TGF-β3 and FGF-2 participated in the angiogenesis of SC pathogenesis. The higher expression of *VEGF-A* accorded with the phenomenon of increased quantity of blood vessels in SC synovium (Fig. 1b, Fig. 2a and b), as VEGF-A is well-known to be mitogenic for endothelial cells^27^ and to regulate vascular permeability^28^.

Then, we investigated the interaction between TGF-β3 and FGF-2. Interestingly, TGF-β3 could not influence the *FGF-2* expression but FGF-2 could upregulate the *TGF-β3* expression. Previously, we showed that the stimulation of FGF-2 on SC synoviocytes upregulated the expressions of chondrogenic and osteogenic genes^26^. Therefore, we concluded that both TGF-β3 and FGF-2 were involved in the pathogenesis of SC, and that the production of TGF-β3 could be regulated by FGF-2.

The clinical importance and guiding significance of this study should be emphasized. For clinicians, this study provided a possible way to treat SC in early stage. By intra-articular injection of the medicine that can suppress the production and activity of TGF-β3, the cartilaginous ECM accumulation process may be stopped. By intra-articular injection of the medicine that can suppress angiogenesis, the nutrient supply may be cut off. Therefore, using these two ways, the formation of LBs may be restrained. For researchers, this study demonstrated the basic structures and features of the synovium and LB of SC. Future researchers may follow the ideas of chondrogenic differentiation of MSCs and the angiogenesis to choose related new cytokines or proteins to make further investigations. Researchers of cartilage tissue-engineering field may also be benefit from the investigations of SC, because by using the chondrogenic cytokines involved in the pathogenesis of SC, it may be possible for them to engineer the artificial cartilage with more similar biological and mechanical properties with natural cartilage.

## Methods

### Samples

All methods were carried out in accordance with the approved guidelines and regulations of the Ethics Committee of School & Hospital of Stomatology, Wuhan University. All experimental protocols were approved by the Ethics Committee of School & Hospital of Stomatology, Wuhan University. Patients were fully informed, and written consents were acquired.

LB specimens and synovium tissues were obtained from three SC patients (Patient No. 1: female, 54 y, left, surgery in May, 2013; Patient No. 2: female, 49 y, left, surgery in July, 2013; Patient No. 3: female, 71 y, right, surgery in September, 2013). The control synovium specimens were acquired from three patients who were subjected to the surgery of open reduction for condylar fracture. The information of these three patients was listed as follows: Patient No. 1: male, 14 y, left, surgery in May, 2013; Patient No. 2: female, 27 y, right, surgery in June, 2013; Patient No. 3: female, 20 y, right, surgery in July, 2013. SC and control synovium specimens were harvested from the region facing joint space.

### Histological and IHC observation

LBs and synovium specimens were fixed in 4% paraformaldehyde solution. For decalcification, LBs were immersed in a solution containing 10% ethylene diamine tetraacetic acid (EDTA) for 3 months. After a series of classic treatments for histological observations, the paraffin-embedded sections of 4 μm-thick were acquired, and disposed with haematoxylin and eosin (HE) staining.

Streptavidin-peroxidase conjugated method was applied for IHC observations, as described previously^29^. Antigen was retrieved using pepsin (DIG-3009, Maixin, China) at 37°C for 30 min. Rabbit-originated antibodies against CD34 (1:400, ZA-0550, Zhongshan Golden Bridge Biotechnology Co., Ltd., China), human TGF-β3 (1:200, 18942-1-AP, Proteintech), and human FGF-2 (1:500, ZS-79, Zhongshan Golden Bridge Biotechnology Co., Ltd., China) were used as primary antibodies and incubated at 4°C for 18 h. The histological sections were then stained by the anti-rabbit streptavidin-peroxidase kit (SP-9001, Zhongshan Golden Bridge Biotechnology Co., Ltd., China). Finally, color development was achieved by reacting with 3, 3′-diaminobenzidine (DAB, 0031, Maixin, China). Hematoxylin was used for counterstaining.

### RT-PCR for comparing SC and normal synovium

To investigate the mRNA expressions of *TGF-β1*, *TGF-β3*, *FGF-2*, *VEGF-A*, *Sox9*, *Wnt-4*, *Runx2* and *Foxc2*, RT-PCR assay was performed. The primer sequences of the above targets were listed in Table 1. As described previously^30^,^31^, SC and control synovium specimens were powdered in liquid nitrogen. According to the instructions of the manufacturer, trizol reagent (Invitrogen, CA, USA) was used to extract the total RNA from the specimens. Reverse transcriptase and oligo dTs were used to achieve synthesizing the cDNA with the assistance of ReverTra Ace kit (Toyobo, Osaka, Japan). The RT-PCR assay was conducted in triplicate using the following protocols: pre-incubation for 1 min at 95°C, followed by 40 PCR cycles for 15 s at 95°C, 20 s at 58°C, and 20 s at 72°C. Electrophoresis was performed subsequently in 1.5% agarose gel containing ethidium bromide. The products were visualized under ultraviolet light, and photographed. The densitometric value of DNA bands was measured with the image analysis software (NIH Image J).

### Cell culture

Cell culture was performed as described previously^32^. Briefly, the SC and control synovium specimens were washed with phosphate buffered solution (PBS) containing penicillin and streptomycin. Then the synovium specimens were cut into 1 mm^3^, and maintained with a solution containing dulbecco's modified eagle medium (DMEM, SH30022.01B, HyClone®) and 15% fetal bovine serum (FBS, SV30087,HyClone®) in a humidified atmosphere of 5% CO_2_ and 95% air at 37°C. When reaching confluence, the synoviocytes were dissociated by trypsin (SH30042,HyClone®). Then the synoviocytes were cultured with DMEM containing 10% FBS.

### Realtime PCR for evaluate the effects of TGF-β3 on SC synoviocytes

The SC synoviocytes of passage 3^rd^ to 6^th^ were cultured with DMEM containing 10% FBS in a 6-well plate. After reaching 70% confluence, the SC synoviocytes were maintained in the DMEM containing 4% FBS in presence or absence of 10 ng/ml recombinant human TGF-β3 (100-36E, PeproTech, USA) for 6 days. The medium was replaced every two days. Realtime PCR was performed to investigate the gene expression levels of *Col2α1*, *Aggrecan*, *Sox9*, *VEGF-A*, *Wnt-4*, *Wnt-11*, *Runx2*, *Foxc2*, *Col1α1*, and *FGF-2*. The primer sequences of the targets were listed in Table 1.

As described previously^31^, according to the instructions of the manufacturer, trizol reagent was used to extract the total RNA from the specimens. The RevertAid First Strand cDNA Synthesis Kit (K-1622, ReverTra Ace-α) was used to achieve reverse transcribing total RNA into cDNA. The semi-quantitative PCR of cDNA samples were disposed with TOYOBO THUNDERBIRD SYBR qPCR Mix (QPS-201). The realtime PCR assay was conducted in triplicate using the following protocols: pre-incubation for 1 min at 95°C, followed by 40 PCR cycles for 15 s at 95°C, 20 s at 58°C, and 20 s at 72°C. The method of comparative threshold cycle (ΔΔCt) was used to evaluate the expression levels of the desired genes which was normalized by the expression level of β-actin gene measured in each sample.

### The stimulation of FGF-2 on TGF-β3 expression

After reaching 70% confluence, the SC synoviocytes were maintained in the DMEM containing 4% FBS in presence or absence of 10 ng/ml recombinant human FGF-2 (AF-100-18B, PeproTech, USA) for 6 days. Then RNA was extracted and realtime PCR was applied to investigate the expression level of TGF-β3 mRNA.

### ALP activity measurement

SC synoviocytes were cultured in DMEM containing 4% FBS with or without 10 ng/ml TGF-β3 for 6 days. As previously described^33^, the SC synoviocytes were lysed in 1% Triton X-100. The ALP activity was measured with colorimetric ALP Assay Kit (A059-2, Nanjing Jiancheng Bioengineering Institute) in accordance with the instructions of the manufacturer, and standardized to the concentration of total protein by bicinchonininc acid (BCA) Assay (P0012, Beyotime Institute of Biotechnology).

### Statistical analysis

Statistical analyses were conducted using SPSS 11.0.0. All data in this study were shown as mean ± standard deviation. Paired t-test was applied to evaluate the significance. α = 0.05 was designated as the significance levels.

## Conclusion

TGF-β3 was expressed in the synovium and LBs of TMJ SC. TGF-β3 upregulated the expressions of chondrogenic, osteogenic, and angiogenic genes in SC synoviocytes. The *TGF-β3* expression was upregulated by FGF-2. Therefore, TGF-β3 was responsible for the pathogenesis of TMJ SC. Inhibition of the functions of TGF-β3 might be a possible method to control the formation and growing of the cartilaginous nodules in early stage of SC in clinical treatment. By verifying the conjecture that the formation of cartilaginous nodule in synovium of TMJ was the outcome of the chondrogenic differentiation of the synoviocytes, this study provided a basic explanation for this rare clinical disease, and also provided an understanding of cartilage regeneration as well as the blood supply for tissue-engineering field. In future studies, we will do investigations to facilitate the formation of cartilaginous nodules in animal TMJ in order to reflect and further discover the process of pathogenesis of SC.

## Author Contributions

Y.L. and X.L. designed this study together. Y.L. and L.A.E.M. performed all the experiments in this study and wrote this paper. M.D. and X.L. performed the surgeries. H.C. prepared all the figures. W.F. and Q.M. analyzed the data. J.L. and M.D. revised this paper. All authors had reviewed this manuscript. Y.L. and L.A.E.M. contributed equally to this paper.

## Supplementary Material

Supplementary Information Title page and supplementary data

## Figures and Tables

**Table 1:** Primer sequence of mRNA templates

mRNA template		Primer sequence
FGF-2	Sense	5′- AGAGCGACCCTCACATCAAG-3′
	Antisense	5′- TCGTTTCAGTGCCACATAC-3′
TGF-β1	Sense	5′- AAGTGGACATCAACGGGTTC-3′
	Antisense	5′- TGCGGAAGTCAATGTACAGC-3′
TGF-β3	Sense	5′- GGTTTTCCGCTTCAATGTGT-3′
	Antisense	5′- TATAGCGCTGTTTGGCAATG-3′
Sox9	Sense	5′- TACGACTACACCGACCACCA-3′
	Antisense	5′- TCAAGGTCGAGTGAGCTGTG-3′
Wnt-4	Sense	5′- CTAGCCCCGACTTCTGTGAG-3′
	Antisense	5′- AAGCAGCACCAGTGGAATTT-3′
Wnt-11	Sense	5′-TGACCTCAAGACCCGATACC-3′
	Antisense	5′-CGTTGGATGTCTTGTTGCAC-3′
Foxc2	Sense	5′- ATCTCAACCACAGCGGGGAC-3′
	Antisense	5′- AGTTGAACATCTCCCGCACG-3′
Runx2	Sense	5′- TCAACGATCTGAGATTTGTGGG-3′
	Antisense	5′- GGGGAGGATTTGTGAAGACGG-3′
VEGF-A	Sense	5′- AAGGAGGAGGGCAGAATCAT-3′
	Antisense	5′- ATCTGCATGGTGATGTTGGA-3′
Col2α1	Sense	5′-CAATCCAGCAAACGTTCCCA-3′
	Antisense	5′-CAGGCGTAGGAAGGTCATCT-3′
Aggrecan	Sense	5′-AGGTCTCACTGCCCAACTAC-3′
	Antisense	5′-AACACGATGCCTTTCACCAC-3′
Osteocalcin	Sense	5′-CTCACACTCCTCGCCCTATT-3′
	Antisense	5′-AACTCGTCACAGTCCGGATT-3′
Col1α1	Sense	5′-GGCAAAGATGGACTCAACGG-3′
	Antisense	5′-ATCATCAGCCCGGTAGTAGC-3′
β-actin	Sense	5′- AGCGAGCATCCCCCAAAGTT-3′
	Antisense	5′- GGGCACGAAGGCTCATCATT-3′

## References

[b1] Tojyo I., Yamaguti A., Ozaki H., Yoshida H. & Fujita S. The expression of fibroblast growth factor receptor-3 in synovial osteochondromatosis of the temporomandibular joint. Arch. Oral Biol. 49, 591–594 (2004). 15126141 10.1016/j.archoralbio.2003.12.009

[b2] Goizueta-Adame C. C. & González-García R. Synovial chondromatosis of the temporomandibular joint: report of 2 patients whose joints were reconstructed with costochondral graft and alloplastic prosthesis. Brit. J. Oral Max. Surg. 48, 374–377 (2010). 10.1016/j.bjoms.2009.07.024 19709788

[b3] Sato J., Notani K.-I., Goto J., Shindoh M. & Kitagawa Y. Synovial chondromatosis of the temporomandibular joint accompanied by loose bodies in both the superior and inferior joint compartments: case report. Int. J. Oral Max. Surg. 39, 86–88 (2010). 10.1016/j.ijom.2009.07.012 19683416

[b4] Chen M.-J. *et al.* Synovial chondromatosis in the inferior compartment of the temporomandibular joint: different stages with different treatments. J. Oral Maxil. Surg. 70, e32–e38 (2012). 10.1016/j.joms.2011.07.014 22033448

[b5] Mikami T. *et al.* Three Case Reports of Synovial Chondromatosis of Temporomandibular Joint: Histopathologic Analyses of Minute Cartilaginous Loose Bodies From Joint Lavage Fluid and Comparison With Phase II and III Cases. J. Oral Maxil. Surg. 70, 2099–2105 (2012). 10.1016/j.joms.2011.09.016 22177806

[b6] Wake M. *et al.* Up-regulation of interleukin-6 and vascular endothelial growth factor-A in the synovial fluid of temporomandibular joints affected by synovial chondromatosis. Brit. J. Oral Max. Surg. 51, 164–169 (2013). 10.1016/j.bjoms.2012.03.004 22475366

[b7] Ravindran S. *et al.* Changes of chondrocyte expression profiles in human MSC aggregates in the presence of PEG microspheres and TGF-β3. Biomaterials 32, 8436–8445 (2011). 21820171 10.1016/j.biomaterials.2011.07.056 PMC3176960

[b8] Morille M. *et al.* New PLGA-P188-PLGA matrix enhances TGF-β3 release from pharmacologically active microcarriers and promotes chondrogenesis of mesenchymal stem cells. J. Control Release 170, 99–110 (2013). 23648834 10.1016/j.jconrel.2013.04.017

[b9] Zheng D. *et al.* Controlled chondrogenesis from adipose-derived stem cells by recombinant transforming growth factor-beta3 fusion protein in peptide scaffolds. Acta Biomater. 11, 191–203 (2015). 25257317 10.1016/j.actbio.2014.09.030

[b10] Park J. S. *et al.* Chondrogenic potential of stem cells derived from amniotic fluid, adipose tissue, or bone marrow encapsulated in fibrin gels containing TGF-β3. Biomaterials 32, 8139–8149 (2011). 21840589 10.1016/j.biomaterials.2011.07.043

[b11] De Bari C., Dell'Accio F., Tylzanowski P. & Luyten F. P. Multipotent mesenchymal stem cells from adult human synovial membrane. Arthritis Rheum. 44, 1928–1942 (2001). 11508446 10.1002/1529-0131(200108)44:8<1928::AID-ART331>3.0.CO;2-P

[b12] Fan J., Varshney R. R., Ren L., Cai D. & Wang D.-A. Synovium-derived mesenchymal stem cells: a new cell source for musculoskeletal regeneration. Tissue Eng. Part B Rev. 15, 75–86 (2009). 19196118 10.1089/ten.teb.2008.0586

[b13] Fujita S., Iizuka T., Yoshida H. & Segami N. Transforming growth factor and tenascin in synovial chondromatosis of the temporomandibular joint: Report of a case. Int. J. Oral Max. Surg. 26, 258–259 (1997). 10.1016/s0901-5027(97)80862-4 9258713

[b14] Sato J., Segami N., Suzuki T., Yoshitake Y. & Nishikawa K. The expression of fibroblast growth factor-2 and fibroblast growth factor receptor-1 in chondrocytes in synovial chondromatosis of the temporomandibular joint. Report of two cases. Int. J. Oral Max. Surg. 31, 532–536 (2002). 10.1054/ijom.2002.0248 12418570

[b15] Yoshida H., Tsuji K., Oshiro N., Wato M. & Morita S. Preliminary report of Ki-67 reactivity in synovial chondromatosis of the temporomandibular joint: An immunohistochemical study. J. Craniomaxillofac. Surg. 41, 473–475 (2011). 22196739 10.1016/j.jcms.2011.10.018

[b16] Bian L. *et al.* Enhanced MSC chondrogenesis following delivery of TGF-β3 from alginate microspheres within hyaluronic acid hydrogels in vitro and in vivo. Biomaterials 32, 6425–6434 (2011). 21652067 10.1016/j.biomaterials.2011.05.033 PMC3134110

[b17] Barry F., Boynton R. E., Liu B. & Murphy J. M. Chondrogenic differentiation of mesenchymal stem cells from bone marrow: differentiation-dependent gene expression of matrix components. Exp. Cell Res. 268, 189–200 (2001). 11478845 10.1006/excr.2001.5278

[b18] Guijarro-Martínez R. *et al.* Bilateral synovial chondromatosis of the temporomandibular joint. J. Craniomaxillofac. Surg. 39, 261–265 (2011). 20605727 10.1016/j.jcms.2010.04.016

[b19] Dijkgraaf L. C., de Boni L. G. M., Boering G. & Liem R. S. B. Structure of the normal synovial membrane of the temporomandibular joint: a review of the literature. J. Oral Maxillofac. Surg. 54, 332–338 (1996). 8600242 10.1016/s0278-2391(96)90755-7

[b20] Nozawa-Inoue K. *et al.* Synovial membrane in the temporomandibular joint-Its morphology, function and development. Arch. Histol. Cytol. 66, 289–306 (2003). 14692685 10.1679/aohc.66.289

[b21] Hardingham T. E., Oldershaw R. A. & Tew S. R. Cartilage, SOX9 and Notch signals in chondrogenesis. J. Anat. 209, 469–480 (2006). 17005019 10.1111/j.1469-7580.2006.00630.x PMC2100356

[b22] Quintana L., zur Nieden N. I. & Semino C. E. Morphogenetic and regulatory mechanisms during developmental chondrogenesis: new paradigms for cartilage tissue engineering. Tissue Eng. Part B Rev. 15, 29–41 (2008). 19063663 10.1089/ten.teb.2008.0329 PMC2817664

[b23] You W. *et al.* Foxc2 regulates osteogenesis and angiogenesis of bone marrow mesenchymal stem cells. BMC musculoskelet. Disord. 14, 199 (2013). 23815774 10.1186/1471-2474-14-199 PMC3710500

[b24] Lee K.-S., Hong S.-H. & Bae S.-C. Both the Smad and p38 MAPK pathways play a crucial role in Runx2 expression following induction by transforming growth factor-beta and bone morphogenetic protein. Oncogene 21, 7156–7163 (2002). 12370805 10.1038/sj.onc.1205937

[b25] Nakanishi S. *et al.* Bone morphogenetic proteins are involved in the pathobiology of synovial chondromatosis. Biochem. Biophys. Res. Commun. 379, 914–919 (2009). 19138670 10.1016/j.bbrc.2008.12.170

[b26] Li Y. *et al.* Fibroblast growth factor 2 involved in the pathogenesis of synovial chondromatosis of temporomandibular joint. J. Oral Pathol. Med. 43, 388–394 (2013). 24372705 10.1111/jop.12146

[b27] Sato J., Segami N., Yoshitake Y. & Nishikawa K. Correlations of the expression of fibroblast growth factor-2, vascular endothelial growth factor, and their receptors with angiogenesis in synovial tissues from patients with internal derangement of the temporomandibular joint. J. Dent. Res. 82, 272–277 (2003). 12651930 10.1177/154405910308200406

[b28] Kumagai K. *et al.* The levels of vascular endothelial growth factor in the synovial fluid correlated with the severity of arthroscopically observed synovitis and clinical outcome after temporomandibular joint irrigation in patients with chronic closed lock. Oral Surg. Oral Med. Oral Pathol. Oral Radiol. Endod. 109, 185–190 (2010). 20034821 10.1016/j.tripleo.2009.09.009

[b29] Zhang R., Wang L. & Peng B. Activation of p38 mitogen-activated protein kinase in rat periapical lesions. J. Endod. 34, 1207–1210 (2008). 18793921 10.1016/j.joen.2008.07.004

[b30] Li J. *et al.* Regulation of HAS expression in human synovial lining cells of TMJ by IL-1β. Arch. Oral Biol. 53, 60–65 (2008). 17868639 10.1016/j.archoralbio.2007.07.011

[b31] Gong Z. *et al.* Use of synovium-derived stromal cells and chitosan/collagen type I scaffolds for cartilage tissue engineering. Biomed. Mater. 5, 055005 (2010). 20826911 10.1088/1748-6041/5/5/055005

[b32] Nagai H. *et al.* Isolation and characterization of synovial cells from the human temporomandibular joint. J. Oral Pathol. Med. 35, 104–110 (2006). 16430741 10.1111/j.1600-0714.2006.00369.x

[b33] Kemmis C. M., Vahdati A., Weiss H. E. & Wagner D. R. Bone morphogenetic protein 6 drives both osteogenesis and chondrogenesis in murine adipose-derived mesenchymal cells depending on culture conditions. Biochem. Biophys. Res. Commun. 401, 20–25 (2010). 20816936 10.1016/j.bbrc.2010.08.135

