# Supplementary material for: Transforming growth factor beta 3 involved in the pathogenesis of synovial chondromatosis of temporomandibular joint
Source: Sci Rep. 2015 Mar 6;5:8843. doi: 10.1038/srep08843 (PMC4351526; doi:10.1038/srep08843)
Supplement: Supplementary Information — Title page and supplementary data [file srep08843-s1.pdf]

Title：

Transforming growth factor beta 3 involved in the pathogenesis of synovial chondromatosis of temporomandibular joint

Author list：

Yingjie Li
Loaye Abdelaziz El.Mozen
Hengxing Cai
Wei Fang
Qinggong Meng
Jian Li
Mohong Deng
Xing Long

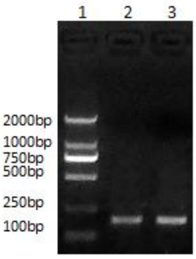

1. Marker
2. Control TGF-β1
3. SC TGF-β1

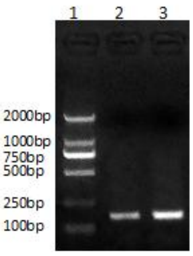

1. Marker
2. Control TGF-β3
3. SC TGF-β3

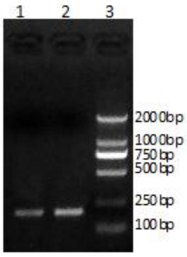

1. Control FGF-2
2. SC FGF-2
3. Marker

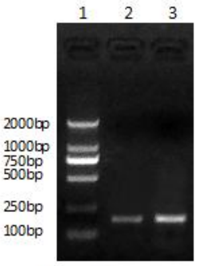

1. Marker
2.Control Sox9
3. SC Sox9

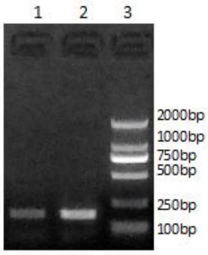

1. Control Wnt-4
2. SC Wnt-4
3. Marker

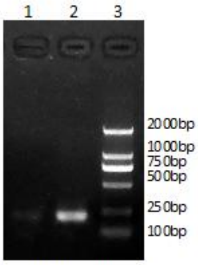

1. Control VEGF-A
2. SC VEGF-A
3. Marker

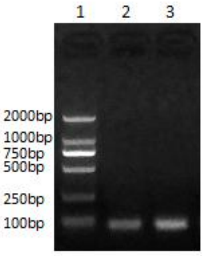

1. Marker
2. Control Foxc2
3. SC Foxc2

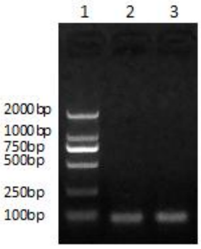

1. Marker
2. Control Runx2
3. SC Runx2

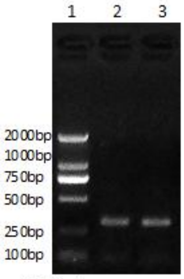

1. Marker
2. Control β-actin
3. SC β-actin
